# Clinical Medicine

**Published:** 1893-04

**Authors:** J. A. Tomhagen

**Affiliations:** Philadelphia, Pa.


### CLINICAL MEDICINE.

J. A. Tomhagen, M. D., Philadelphia, Pa.

Case I.—Was called to see Mrs. E. P., æt. fifty-one, and mother of thirteen children. Had itch nine years ago, suppressed with ointment, and measles eight years ago. At present, life is a burden to her. Says she does not care to live, if this *urinary trouble* can't be cured. Burning in the feet at night; this is merely a sensation, since her husband says they are actually cold. She has a longing, alternately, for sweet and sour things. Lies on right side with legs drawn up; this is the only position in which she can obtain any sleep. Chilly sensation at times. Leucorrhœa yellowish and again whitish, flowing profusely during the day.

Great burning after urinating. Shooting pain in the tongue, which looks red and angry; *burning down centre of tongue.* Cannot eat fat meat because it brings on diarrhœa. Her menses always delayed a week, lasted six days, but were scanty, flowing in fits and starts. During the week before menses she had sour vomiting and diarrhœa; drawing up and down sensation in abdomen; hot feet and legs when walking. At present, she has frequent urination and passes only a teaspoonful at a time with great bearing down. Urine high colored.

Sept. 20th, 1888.—Puls.$^{10m}$, six powders, one every night and morning for three days.

Nov. 6th.—Considerably better, but I still have the drawing in my stomach (?), and burning in my feet and legs. CM.

Dec. 15th, she writes: "Am almost well, but think I had better continue taking medicine awhile longer."

*Remarks.*—This woman had been in bed for months, off and on, and utterly unable to supervise her domestic affairs.

The strangury was intense, and the chief difficulty for which I was summoned. The patient, however, living twenty miles from my office, could not have me come often, so I thought it advisable to prescribe on the constitutional symptoms, trusting that the remedy selected on *these*, would also cure the *strangury.*

The changeability of the symptom: the symptom she had during her menstrual epoch; the burning sensation down centre of tongue; the aggravations and ameliorations all pointed unmistakably to Puls.

One prescription was sufficient to cause the vicegerent of the soul to re-establish order out of chaos.

Case II.—Mrs. E. T., æt. fifty-seven. Brunette. Migraine seven years. Heavy sore feeling over right eye; sleepy and languid all day; bitter taste in morning and sick at stomach; pain begins in occiput and moves forward to right supra-orbital region; wants to go into a dark room, away from everything and everybody; does not want to hear nor see anything; pain in head relieved by vomiting, which is watery and bilious; yellow coating on base of tongue; "bowels regular;" no appetite during attacks; skin gets yellow when about to have an attack; urine high colored ; dark coffee-ground sediment in urine during attack ; generally feels better in cold weather ; can sit down any time of day and go to sleep ; very thirsty ; photophobia of right eye during attack. These headaches last from one to seven days, beginning about eight or nine A. M. and leaving at seven P. M. Urine becomes clear as headache improves.

Oct. 5th, 1891.—Sang.$^{1600}$.

Oct. 12th.—General improvement.

Last Wednesday, was giddy after dinner, and again slightly this morning, but no headache. CM.

Nov. 4th.—Soreness and stiffness in palate in morning. Scratching and scraping in throat. Chilly sensation ; dull feeling in forehead. Watery discharge from nose and eyes.

Dry cough worse in room.

Discharge worse in room.

General improvement in open air. CM.

Dec. 20th. No headache till December 17th, which was slight and of same nature as old ones. Sang.$^{cm}$.

Dec. 31st.—" I have so much wind in stomach and only after a few mouthfuls." No appetite ; thirstless ; mouth and throat dry in morning ; sensation of load in pit of stomach ; chilly ; costive ; left eye waters ; tired, heavy feeling ; worse from coffee ; better in open air ; sleeps lying on right side ; feet cold ; wants head uncovered always, and thinks she takes cold that way ; urine high colored. Lyc.$^{1400}$.

March 2d.—Was all right till last few days ; some of old symptoms returning. Lyc.$^{71m}$.

March 20th.—On the 17th inst., her *right foot was very cold, while left was normal* (she thought this very strange). Also had the full feeling in stomach, and a pain in left supra-orbital region extending through to back of head. CM.

March 30th.—Reports herself well.

*Remarks.*—The second dose of Sang. produced no change. Gradually symptoms developed calling for Lyc., which finished the cure. I searched all the books to ascertain whether Lyc. followed Sang., because the symptoms so loudly called for it, but I could not find a single confirmation.

